# Seminoma Arising From an Intra-abdominal Testis in a Phenotypic Female With Complete Androgen Insensitivity Syndrome (CAIS): A Rare Case Report From a South Indian Tertiary Cancer Centre

**DOI:** 10.7759/cureus.106608

**Published:** 2026-04-07

**Authors:** K. V. Ramya Lakshmi, Gnanasambandan Ramanathan, Ravi Chandran Ambalathandi

**Affiliations:** 1 Medical Oncology, Sri Ramachandra Institute of Higher Education and Research, Chennai, IND; 2 Division of Medical Research, SRM Institute of Science and Technology, Chennai, IND

**Keywords:** androgen insensitivity syndrome, case report, cytoreductive surgery, germ cell tumors, intra-abdominal testis

## Abstract

Complete androgen insensitivity syndrome (CAIS) is a rare X-linked disorder, which is present in genetically confirmed males who exhibit female phenotypic characteristics. In this case, an intra-abdominal testis, which subsequently transformed into a malignant germ cell tumor, is a rare malignant evolution that is infrequently reported in phenotypically female patients.

A South Indian origin, 41-year-old phenotypic female with untreated primary amenorrhea presented with progressive abdomino-pelvic distension. MRI imaging demonstrated a large pelvic mass with retroperitoneal lymphadenopathy; biopsy confirmed dysgerminoma with elevated beta-human chorionic gonadotropin (β-hCG) and lactate dehydrogenase (LDH). She completed neoadjuvant bleomycin, etoposide, and cisplatin (BEP) chemotherapy followed by cytoreductive surgery, which revealed intra-abdominal testes with a complete pathological response, and subsequent karyotyping confirmed a 46,XY pattern consistent with CAIS.

Malignant transformation of intra-abdominal testicular tissue in a phenotypically female patient is exceptionally rare and emphasizes the critical importance of early diagnosis; a proper treatment regimen is warranted to improve patient care.

## Introduction

Complete androgen insensitivity syndrome (CAIS) is a rare X-linked disorder. A mutation in the androgen receptor (AR) gene, located on the X chromosome (Xq 11-12), is the main aetiology for this disorder [1]. The prevalence of CAIS is between three and five in 100,000 individuals globally, and they are genetically confirmed males (46,XY). However, these individuals phenotypically present as females with breasts and vulva that are usually normal, while body hair could be less pronounced [2]. A recent case report and systematic literature review on androgen insensitivity syndrome by Apollon Karseladze et al. reported a total of 225 CAIS cases globally [3]. The malignant transformation of the gonads is a severe complication of CAIS patients; also, the risk was positively correlated with the age of the CAIS individuals [4]. Despite this androgen insensitivity, the testes produce normal levels of anti-Müllerian hormone (AMH) during fetal development. AMH, secreted by Sertoli cells, induces regression of the Müllerian ducts, which would otherwise develop into the uterus, fallopian tubes, and upper vagina. Consequently, individuals with CAIS lack Müllerian structures, explaining the absence of a uterus and the presence of a shortened or blind-ending vagina despite a female external phenotype. However, CAIS with a testicular tumor (germ cell tumors) is very rare. Here, we present a case of a 41-year-old female who presented with symptoms of abdominal distension. The case was subsequently investigated and diagnosed as a case of androgen insensitivity syndrome, primary amenorrhea, and intra-abdominal testis, which subsequently transformed into a malignant germ cell tumor, a rare malignant evolution that is infrequently reported in phenotypically female patients in our south Indian population, and treatment strategies are discussed.

## Case presentation

**Figure 1:**
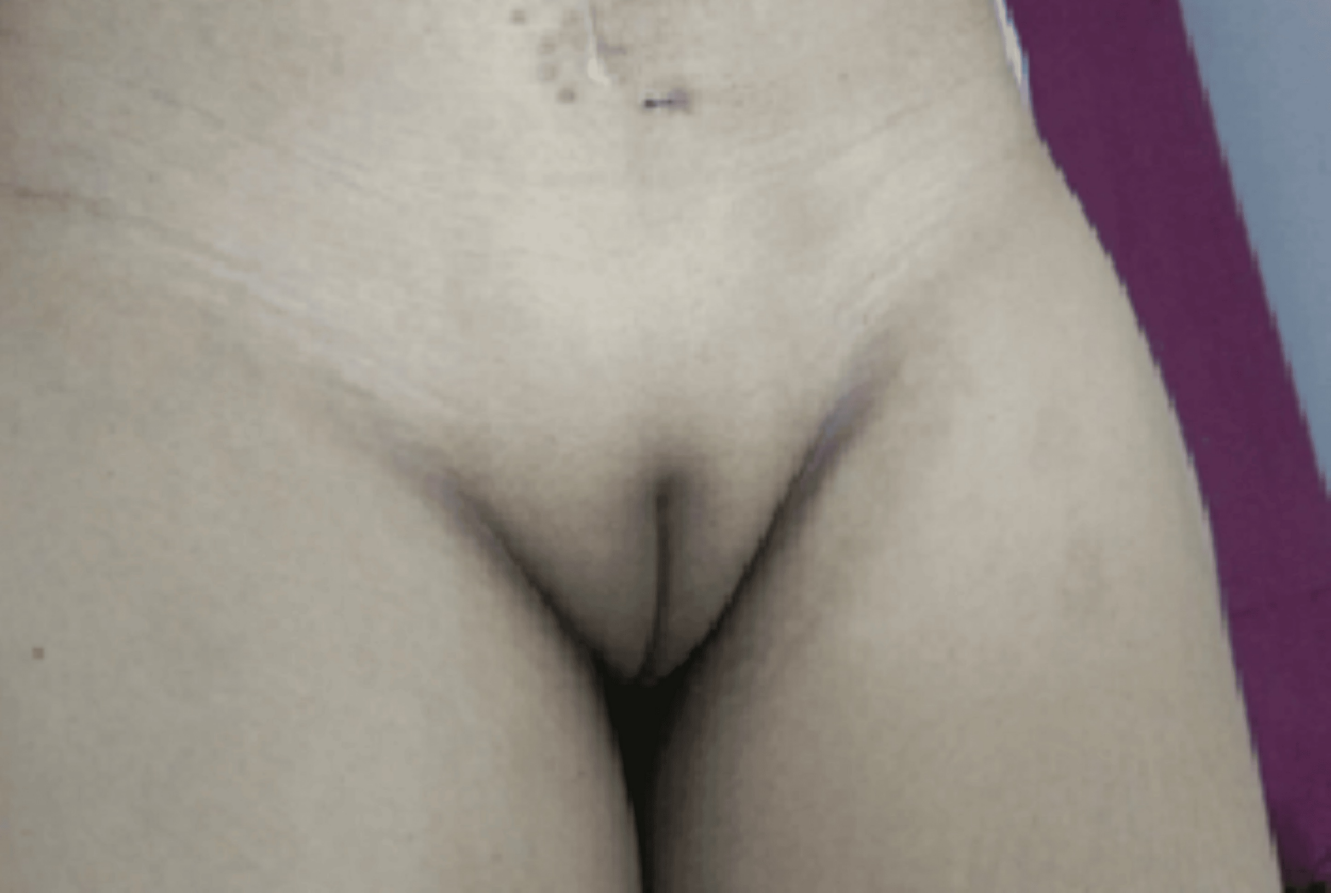
Absence of pubic hair growth

**Figure 2:**
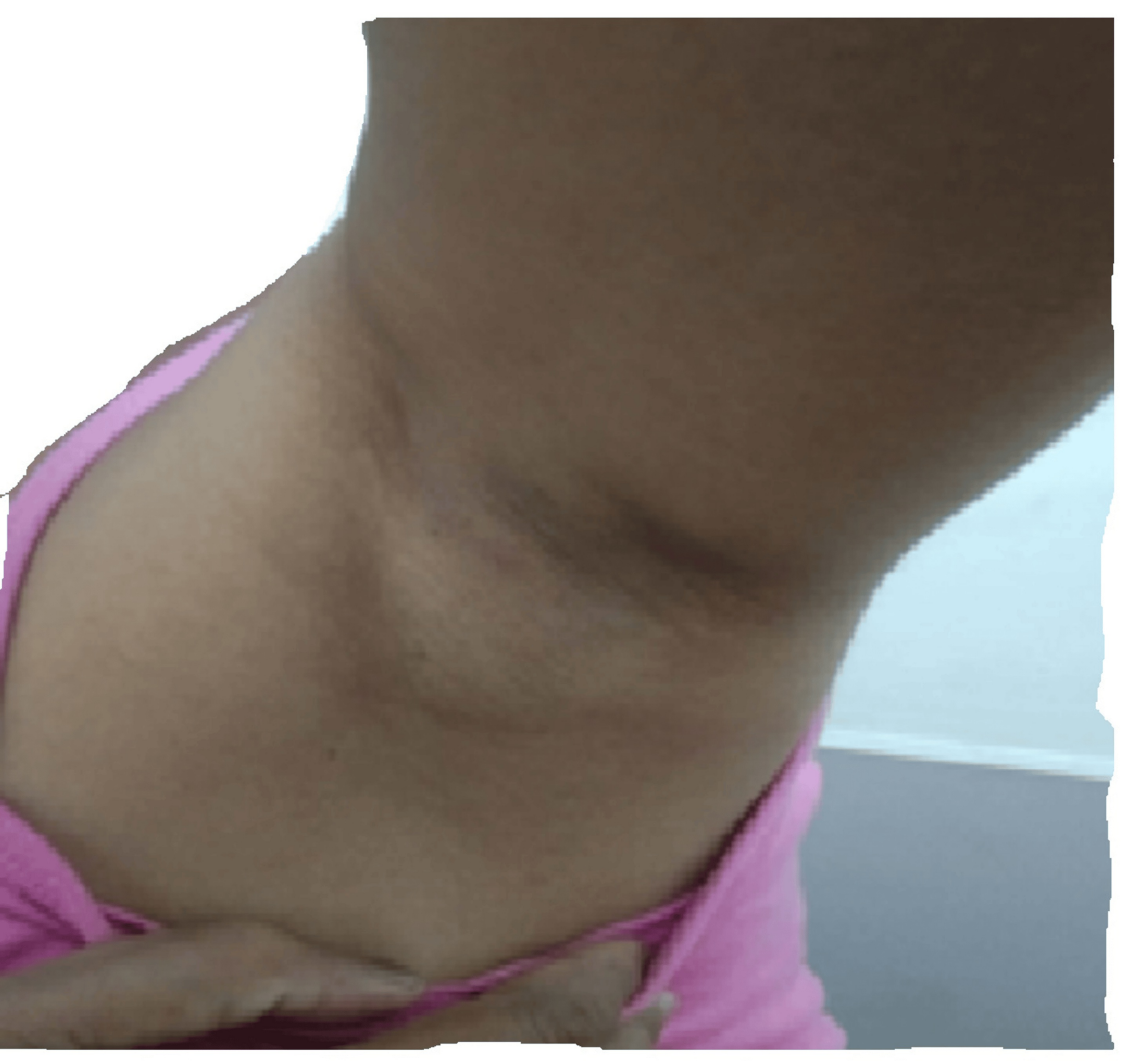
Absence of axillary hair

An unmarried woman with a history of primary amenorrhea was diagnosed with uterine agenesis at the age of 22 years, but she was not evaluated further for the same. Recently, at the age of 41 years, a South Indian origin phenotypically female visited the Department of Medical Oncology at Sri Ramachandra Institute of Higher Education and Research (SRIHER), Chennai, Tamil Nadu, with complaints of lower abdomen pain and distension. This distension had increased six to seven months earlier, accompanied by mild to moderate pain. On examination 12cm x 10cm mass was palpable in the lower abdomen, and the lower border of the mass was not palpable. Further, the patient's genital examination revealed the normal development of external female genitalia with complete absence of pubic hair growth as shown in Figure 1. The patient had normal breast development without axillary hair growth as shown in Figure 2.

**Figure 3:**
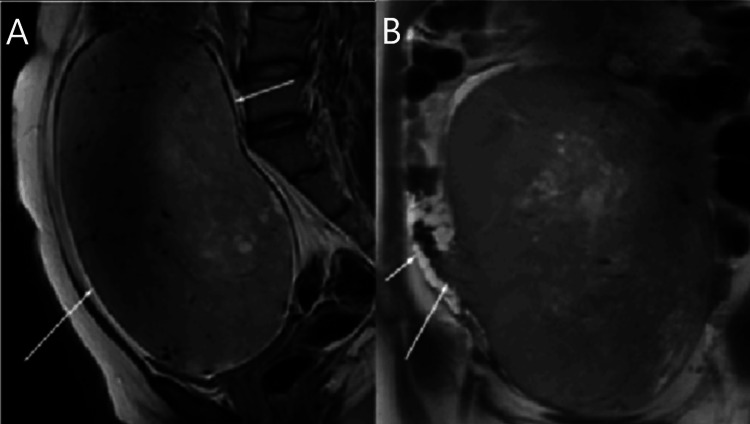
Large abdominopelvic mass of size 12cm x 15cm x 21cm extending up to L4 vertebra and lying close to the anterior abdominal wall MRI abdomen and pelvis (A: Sagittal view, B: Coronal view) revealed a well-circumscribed large abdomino-pelvic lesion of size 12cm x 15cm x 21cm (APxTRANSxCC) extending up to the L4 vertebra and lying close to the anterior abdominal wall.

Imaging with magnetic resonance imaging (MRI) of abdomen and pelvis revealed a well-circumscribed large abdomino-pelvic mass of size 12cm x 15cm x 21cm as described in Figure 3, with retroperitoneal lymphadenopathy, the largest measuring 8.3cm x 5.7 cm. The uterus and right ovary were not visualized, while the left ovary was normal. Ultrasound-guided biopsy from the abdomino-pelvic mass was reported as germ cell tumor - dysgerminoma. Further, the immunohistochemistry (IHC) was also strongly positive for SALL4 and OCT4, and negative for PAX8 and WT1 germ cell tumor markers. Tumor markers showed elevated beta-human chorionic gonadotropin (β-hCG) levels of 65.57 mIU/ml, lactate dehydrogenase (LDH) levels of 1381 U/L, while alpha-fetoprotein (AFP) levels of 2.58 ng/mL and CA125 were normal.

At diagnosis, the disease was staged as TxN3M0S1, consistent with Stage IIC (seminoma/dysgerminoma equivalent). Further, this case was discussed at the multidisciplinary tumor board (SRIHER), and in view of a large primary lesion abutting local structures and retroperitoneal lymphadenopathy, planned for neo-adjuvant chemotherapy with the bleomycin, etoposide, and cisplatin (BEP) regimen followed by reassessment for surgery. She completed four cycles of the BEP regimen, following which there was a significant reduction in abdomino-pelvic mass and retroperitoneal lymph nodes. Restaging scans were performed following four cycles of BEP chemotherapy. MRI of the abdomen and pelvis demonstrated a marked reduction in the primary abdominopelvic mass, along with a significant decrease in retroperitoneal lymphadenopathy. There was no evidence of disease progression. After every cycle, the β-hCG, AFP, and LDH levels were also measured (Table 1). Then the patient was taken up for interval cytoreductive surgery. Surgery was performed four weeks after the last BEP cycle, once blood counts recovered and repeat imaging confirmed maximal response. Tumor markers were not repeated right before surgery. It was repeated before starting the fourth cycle and was within normal limits.

**Table 1:** The measurement of tumor marker levels after every cycle of neo-adjuvant chemotherapy with the bleomycin, etoposide, and cisplatin (BEP) regimen AFP: alpha-fetoprotein, β-hCG: beta-human chorionic gonadotropin, LDH: lactate dehydrogenase

Neo-adjuvant chemotherapy with the BEP regimen	AFP (ng/mL)	β-hCG (mIU/ml)	LDH (U/L)
Cycle 1	2.58	65.57	1381
Cycle 2	1.76	4.47	600
Cycle 3	2.32	5.08	342
Cycle 4	2.73	5.61	221

**Figure 4:**
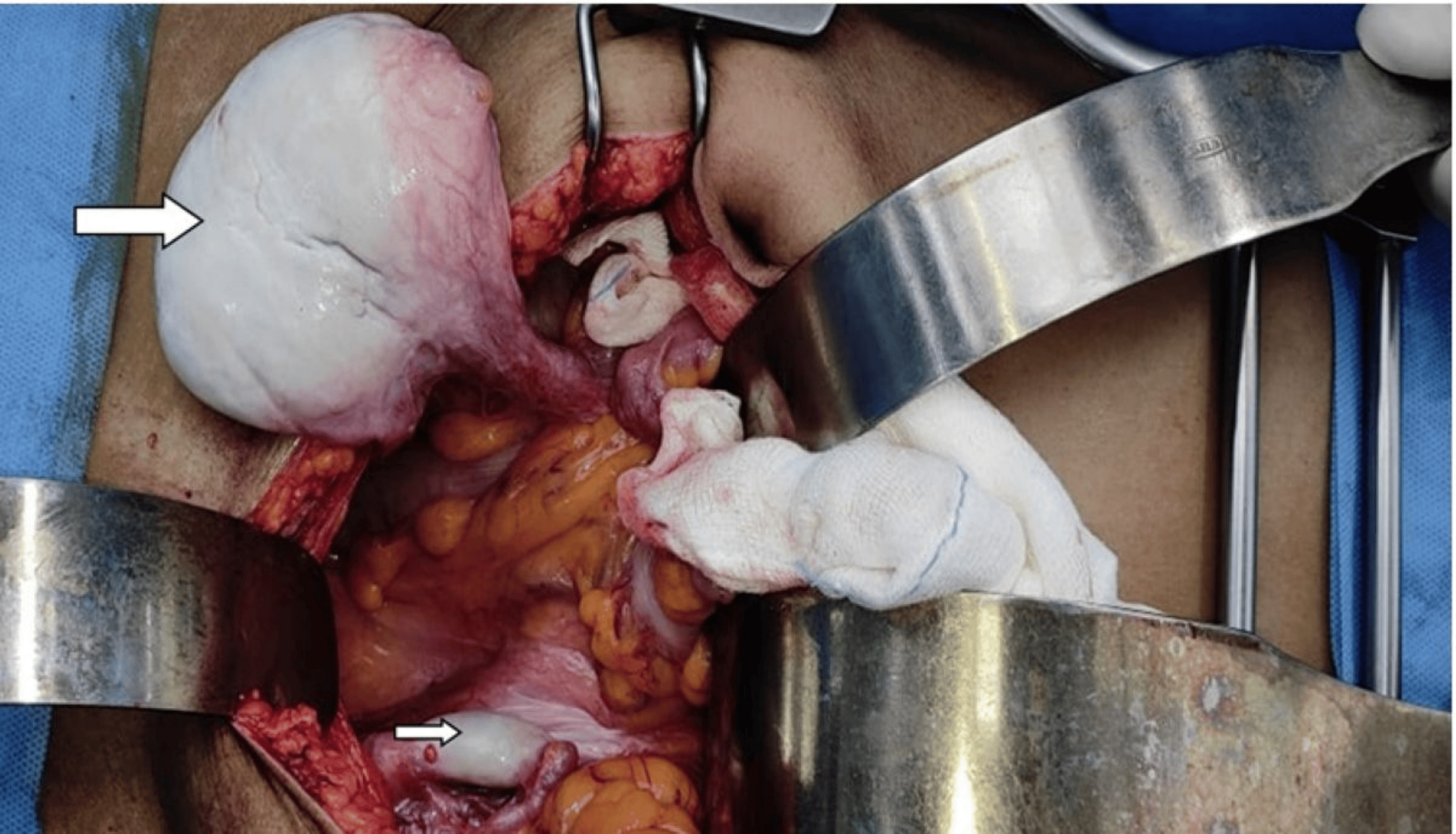
Testicular mass being separated from the local structures. Big arrow: Right testis, Small arrow: Left testis

**Figure 5:**
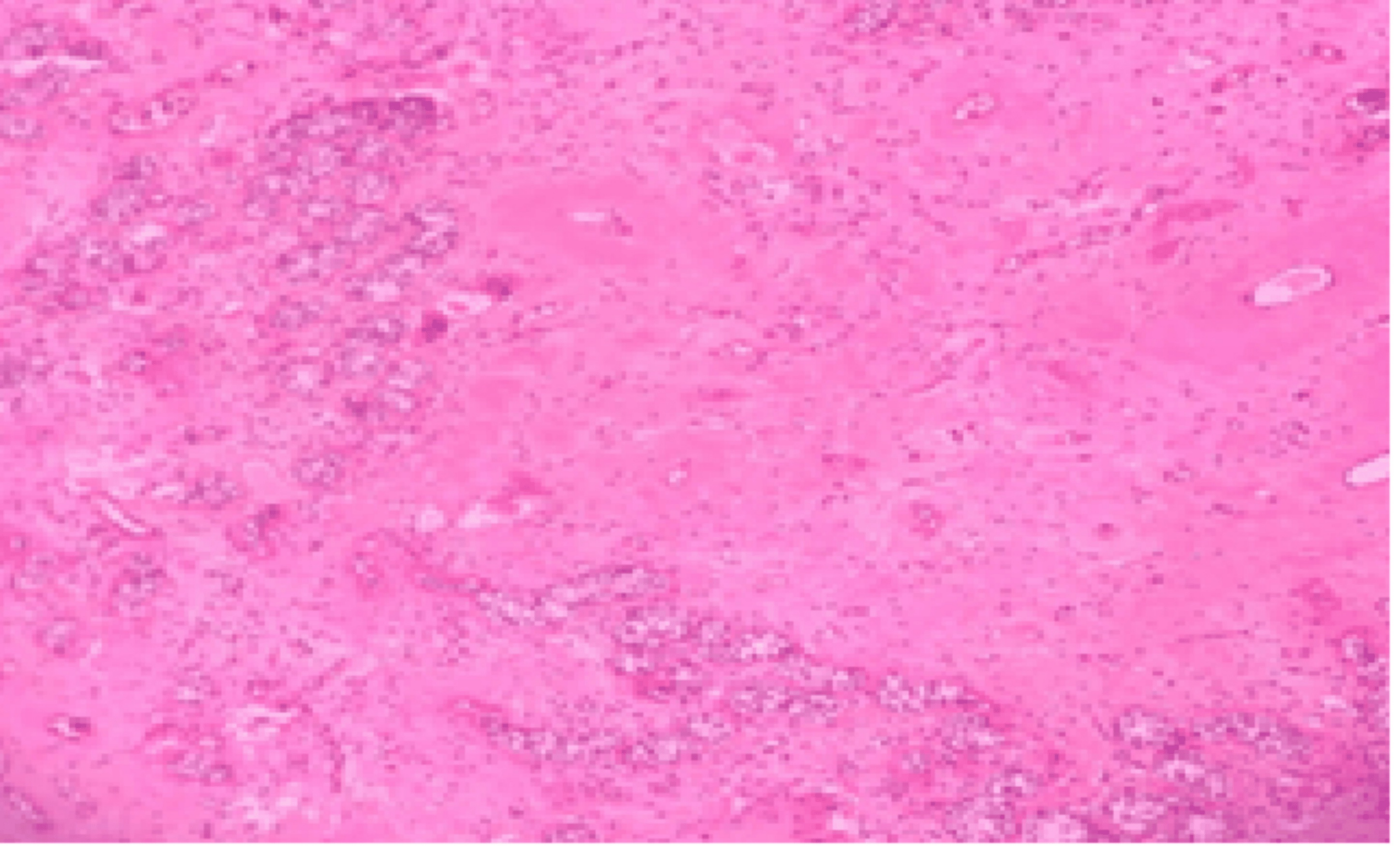
Photomicrograph of right gonad showing fibrosis, inflammatory infiltrate, congested blood vessels and residual tubular structures (H&E stain, 10×)

**Figure 6:**
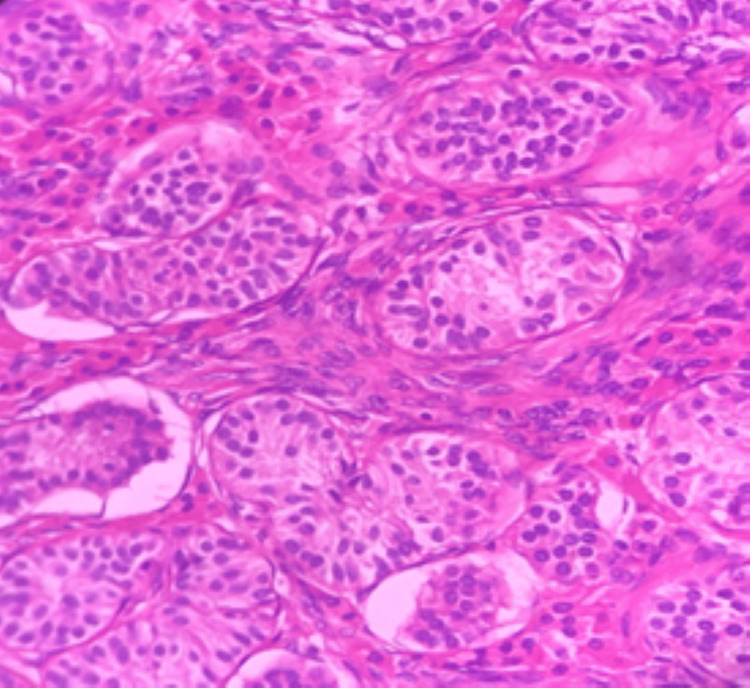
Photomicrograph of left gonad showing well-formed tubular structures (H&E stain, 40×)

**Figure 7:**
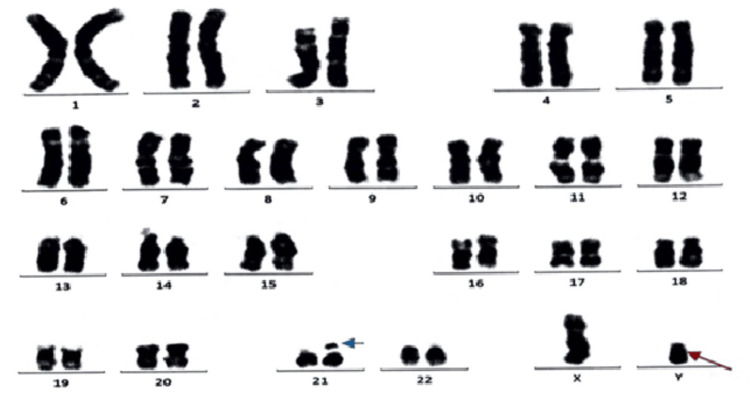
Karyotyping analysis of the patients showing 46,XY The red arrow indicates that the Y chromosome was found along with XX

Intra-operatively, the abdomino-pelvic mass was identified as the right testis with enlarged vas deferens, as shown in Figure 4, with a normal left testis. Post-operative histopathology of the right testicular mass revealed it to be a fibrosis with entrapped residual tubular structures with no residual tumor (pCR), as shown in Figure 5, and the left testis shows a tubular structure with no evidence of malignancy, as shown in Figure 6. Later, karyotyping was done, which showed a 46,XY karyotype (Figure 7), and high testosterone levels of 381.40 ng/dl. Hence, she was diagnosed with complete androgen insensitivity syndrome. Postoperative tumor markers were within normal limits. No adjuvant chemotherapy or radiation was given after surgery because she had already completed four cycles of BEP and there was complete pathologic response. Tumor markers were normalized and there were no viable residual malignant cells identified in the biopsy specimen. In seminoma/dysgerminoma with pCR after chemotherapy and surgery, further treatment is generally not required and surveillance is appropriate. She completed her treatment for seminoma without any significant problems and is now on regular follow-up. History, physical examination and serum tumor markers were done every three months for the first two years, followed by every six months thereafter. CT abdomen/pelvis was done every six months for the first two years followed by annually thereafter. Patient has remained disease-free for about 4.5 to five years from the completion of therapy.

## Discussion

In 1953, John Morris described the ‘testicular feminization syndrome’, later known as CAIS [5]. The primary amenorrhea, 46,XY karyotype, and female phenotype with normal development of breast, external genitalia, and complete inability of the cell to respond to androgens are the major characteristics of CAIS [6]. Further, testes are also reported in the abdomen bilaterally (50%-70% cases) or the inguinal region (20% cases), or located both in the abdomen on one side and in the inguinal region on the other side (10%-30% cases), and may be in the retroperitoneum [7]. Furthermore, a recent case report revealed that 5% of androgen insensitivity syndrome transform into malignancy, with a particularly low prevalence of <1% in CAIS [8].

The diagnosis of CAIS is made at puberty, but this is debatable with clinicians and researchers due to discrepancies in the onset of puberty, either in the early or late stage, compared with a normal 46,XX female, and primary amenorrhea. During birth, the CAIS patients are normal, like other 46,XX female children with normal external features. This is one of the major limitations to early diagnosis of CAIS. Although karyotyping and measuring hormone levels are good methods for early diagnosis, since the CAIS child is normal, like 46,XX at birth, these methods are not being followed for early diagnosis. However, the family history and family pedigree may be conducted based on the genetic disorder running in the family to identify CAIS cases. In this study, the identified CAIS case was at the age of 22, and there was no follow-up by the patient due to the few complications and lack of awareness. By the time the patient reached the clinic, the germ-line malignancy had developed. Therefore, the need for good awareness and family pedigree are recommended for early diagnosis of genetic disorders.

Complete androgen insensitivity syndrome is associated with primary amenorrhea, absence of uterus and upper vagina, undescended testes, infertility, and increased gonadal tumor risk with age. Patients may also experience reduced bone density after gonadectomy, along with psychological and sexual health concerns. Counselling should include clear explanation of the condition (46,XY with androgen resistance), discussion of infertility, and need for lifelong endocrine follow-up. Estrogen replacement, bone density monitoring, and timely gonadectomy are essential. Psychological, sexual, and genetic counselling should be offered, and multidisciplinary care is recommended to support long-term physical and emotional well-being.

There is heterogeneity in the treatment of CAIS malignancy globally; most cases are orchidectomy/gonadectomy in addition to post-operative hormonal therapy, which is followed as treatment. In the current patient, as recommended by the multidisciplinary tumor board of SRIHER, the neo-adjuvant chemotherapy with the BEP regimen for four cycles was followed to shrink the tumor size. Then, cytoreductive surgery was done. Follow-up of the patient showed a significant improvement in the patient's life. Further, to understand the different treatment regimens and diagnosis methods followed in CAIS cases, reports presented in the Indian population were documented in Table 2.

**Table 2:** Complete androgen insensitivity syndrome (CAIS) reported in Indian populations DSD: differences of sex development; PAIS: partial androgen insensitivity syndrome; AR: androgen receptor

Case Report	Number of cases	Patients Information	Diagnosis	Karyotyping	Treatment
Gwendolyn Fernandes, 2022 [9] Case series	8	46,XY DSD cases - CAIS/testicular feminising syndrome (n=8). Seminiferous tubules are lined mainly by Sertoli cells and a few germ cells; no spermatogenesis. Immature/hypoplastic testis on histology. Benign cysts on the testis.	CAIS/Testicular Feminising Syndrome	46,XY DSD	Surgical genital reconstruction, prophylactic gonadectomies, and hormonal supplementation
Mukhopadhyay et al., 2021 [10]	3	Case 1: A 16-year-old female; MRI of the pelvis revealed a vagina measuring 5 cm in length, and a Gartner’s duct cyst was seen in the posterior vaginal wall. Bilateral testes were seen along the lateral pelvic walls. Bilaterally suggestive of Sertoli cell adenomas. Case 2: A 15-year-old female; MRI imaging of the pelvis showed bilateral undescended small testes. There were paratesticular cysts seen bilaterally. A small penile shaft with corpora cavernosa and bilateral seminal vesicles was also seen, along with a rudimentary vagina. Loss of germ cells in the tubules with immature Sertoli cells and Leydig cell hyperplasia. Case 3: A 23-year-old female; MRI showed bilateral intra-abdominal testes. The penile structure or phallus was not identified. Seminiferous tubules lined by Sertoli cells, with 50% showing luminal formation and Leydig cell hyperplasia.	CAIS	46,XY	Laparoscopic gonadectomy and postoperatively, they were started on oestrogen therapy.
Ram et al., 2021 [11]	1	42-year-old female; absence of uterus, cervix, and fallopian tube, with the presence of right ovary in the right inguinal canal and left ovary in the intra-abdominal region. A testis-like structure was detected in the right inguinal canal	CAIS	46,XY	Gonadectomy with herniotomy was done.
Thirunavukkarasu et al., 2016 [12]	1	17-year-old girl; ultrasonography revealed an absent uterus, hypoplastic vagina, and well-defined mass lesions inguinal hernia at the age of five years. Primary amenorrhoea Sertoli cell adenoma.	CAIS	46,XY	Surgical removal of the gonads. Postoperative follow-up and hormone supplementation.
Raina et al., 2018 [13]	1	16‑year‑old; bilateral inguinal swellings measuring 4 cm × 2 cm and 2 cm × 1 cm. The external genitalia were underdeveloped. Ultrasonography of the pelvic and inguinal region revealed the absence of the uterus, fallopian tubes, and ovaries. Bilateral testis in the inguinal region. Bilateral Sertoli cell adenoma along with unilateral serous cyst.	CAIS	46,XY	Gonadectomy.
Arora et al., 2018, [8]	1	35‑year‑old married female; malignant transformation of the undescended testis. The left gonad was not separately palpable. The uterus was absent. The right gonad was present in the right iliac fossa. The histopathology confirmed the presence of seminoma.	CAIS	46,XY	Laparotomy with debulking of the tumor and pelvic lymphadenectomy.
Arya et al., 2021, [2] The cohort study included 150 cases	6 CAIS	All CAIS were reared as females and 83.3% of PAIS as males with no gender dysphoria. Metastatic dysgerminoma was seen in one patient in CAIS, while none in the PAIS group had malignancy. Fifteen different (including six novel) pathogenic/likely pathogenic variants in AR were found. Nonsense and frameshift variants exclusively led to the CAIS phenotype.	CAIS	46,XY DSD	NA
Babulreddy Hanmayyagari, 2015, [14]	1	20-year-old female; Local examination was normal with a blind vagina, about 3x2cm palpable inguinal masses felt bilaterally. External genitalia were like normal female at birth, with small inguinal swellings bilaterally. Ultrasound abdomen no Mullerian structures.	CAIS	46,XY	Bilateral orchidectomy and vaginoplasty. Postoperatively, on estrogen replacement therapy.

## Conclusions

Although the CAIS is very rare, it's distressing to individuals as well as family members. It requires expert psychological handling in collaboration with an oncology surgeon, gynecologist, psychiatrist specialist and proper genetic counselling to combat CAIS. Prenatal screening and prenatal genetic diagnosis with advanced multi-omics sequencing methods are suggested to identify this CAIS in early stages. Further, proper genetic counselling is important for long-term care with CAIS. These advanced technologies may provide a case-based etiology for a better understanding of disease pathophysiology and treatment regimen, which ultimately increases the patient's quality of life.
